# The impact of the Lab4 probiotic on neurodegenerative processes in a murine Alzheimer’s disease model

**DOI:** 10.3389/fnins.2026.1791299

**Published:** 2026-03-31

**Authors:** Timothy R. Hughes, Thomas S. Webberley, Daniel John, Sophie Thomas, Joshua Kerry-Smith, Daryn R. Michael, Ryan J. Bevan, James E. Morgan, Sue Plummer

**Affiliations:** 1 Systems Immunity Research Institute, School of Medicine, Cardiff University, Cardiff, United Kingdom; 2 Cultech Ltd., Baglan Industrial Park, Neath Port Talbot, United Kingdom; 3 UK Dementia Research Institute, Cardiff University, Cardiff, United Kingdom; 4 School of Optometry and Vision Sciences, Cardiff University, Cardiff, United Kingdom

**Keywords:** Alzheimer’s disease, cognition, inflammation, microbiota, neurodegeneration, probiotics

## Abstract

**Introduction:**

The gut-brain-axis is increasingly recognised as a mediator of neurodegenerative processes, with the gut microbiota emerging as a potential target for intervention. The Lab4 probiotic has demonstrated neuroprotective activity *in vitro* and, here, we have investigated its impact on aspects of neurodegeneration in the 3xTg Alzheimer’s disease (AD) murine model.

**Method:**

Male 3xTg-AD mice were fed a high fat diet (to accelerate neurodegeneration) with or without daily Lab4 probiotic supplementation for 84 days. Endpoints included hippocampal neuronal spine density, novel object recognition, whole-brain gene expression, plasma cytokines/lipids, body weight, and faecal microbiota composition.

**Results:**

Lab4 Probiotic supplementation preserved the neuronal spine density, particularly thin spines, and improved recognition memory. Gene expression analysis of whole brain extracts detected reductions in pro-inflammatory markers (IL-5 and Caspase-1) and plasma analysis revealed reduced levels of pro-inflammatory TNF-*α*. The probiotic also mitigated weight gain, though plasma lipid profiles were unchanged. Microbiota analysis indicated increased abundance of *Blautia* and decreased *Muribaculaceae* in probiotic-supplemented mice, alongside reduced numbers of viable yeast.

**Discussion:**

These preliminary findings highlight a neuroprotective impact in 3xTg-AD mice receiving the Lab4 probiotic and warrant more extensive assessments in murine models and/or human subjects.

## 1 Introduction

Neurodegeneration underlies several chronic neurological conditions, including Alzheimer’s disease (AD), Parkinson’s disease, and Huntington’s disease, and these are characterised by the progressive deterioration of neuronal structure and function, ultimately leading to neuronal death and impairment of cognition, movement, and other brain functions (Gadhave et al., 2024). Neurodegeneration can be exacerbated by conditions such as obesity, chronic systemic inflammation and/or oxidative stress (Wang, 2024) but despite prolonged research, there are no preventative/curative treatments available for AD.

The gut microbiota can provide neuroprotective metabolites such as short chain fatty acids (SCFAs) and help maintain the integrity of the intestinal lining to prevent leakage but the factors that contribute to neurodegeneration can also disrupt the balance of the gut microbiota (Loh et al., 2024). There is growing interest in the use of probiotics (‘live microorganisms which, when administered in adequate amounts, confer a health benefit on the host’ (Hill et al., 2014) to restore the balance of the microbiota as a means of allaying the neurodegenerative pathway (Kim et al., 2025; Tabaza and Hartman, 2025). A study in the 3xTg Alzheimer’s Disease (AD) mouse model with a multistrain probiotic reported reductions in progression of AD (Bonfili et al., 2017) and decreases in the progression of this neurodegenerative disease have been seen with lactobacillus based formulation also in the 3xTg model (Medeiros et al., 2024). Furthermore, we have observed neuroprotective effects with probiotics in the same model (Webberley et al., 2022; Webberley et al., 2023).

The Lab4 probiotic, comprising *Lactobacillus acidophilus* CUL21, *Lactobacillus acidophilus* CUL60, *Bifidobacterium bifidum* CUL20 and *Bifidobacterium animalis* subsp. *lactis* CUL34, has been shown to impart *in vitro* protective effects on neuronal cells (Michael et al., 2019). In this study, we follow up on these results, looking at the impact of Lab4 on the progression of neurodegeneration and cognitive decline in metabolically challenged (by means of a high fat diet) 3xTg mice.

## 2 Methods

### 2.1 Husbandry and study design

All experimental procedures were carried out under the United Kingdom Home Office Project License (P8159A562) by designated and trained licensees; 20 3xTG-AD male mice (3 months old) were housed in individual Scantainers (two–four mice per cage) and randomly assigned into two groups: 10 mice received high fat diet (HFD) containing 21% (w/w) pork lard with 0.15% (w/w) cholesterol (SDS, Witham, United Kingdom; product code: 821424) for 84 days (termed the ‘*HFD*’ group) and the other 10 mice received HFD supplemented with a daily dose of 5 × 10^8^ colony forming units (CFU)/mouse/day of the Lab4 probiotic (a human equivalent dose of 5 × 10^10^ CFU/day; Nair and Jacob, 2016) comprising *Lactobacillus acidophilus* CUL21, *Lactobacillus acidophilus* CUL60, *Bifidobacterium bifidum* CUL20 and *Bifidobacterium animalis* subsp. *lactis* CUL34 for 84 days (termed the ‘*HFDP*’ group). Freeze-dried probiotic preparations were mixed into HFD and weekly batches were stored at 4 °C prior to administration to the mice. Mouse body weights were monitored fortnightly throughout the intervention period. At the endpoint (day 84) mice were terminated by CO_2_ inhalation. The results for the *HFD* control mice have been reported previously (Webberley et al., 2022).

### 2.2 Sample collection

Faecal pellets were collected from each mouse at the end of the study and stored at −80 °C. Blood samples were collected termination via cardiac exsanguination into a heparinised microfuge tube. Plasma was separated by centrifugation at 3,000 x g for 5 min. Plasma was stored at −80 °C before use. Whole brains were extracted and separated into half hemispheres. The right hemispheres were transferred into phosphate buffered saline (PBS) for immediate hippocampal spine quantification and the left hemispheres were snap frozen and stored at −80 °C prior to gene expression analysis.

### 2.3 Hippocampal spine quantification

The quantification of thin, stubby and mushroom spine densities on hippocampal neurones was performed as previously described using the Filament Tracer module in Bitplane Imaris software (v9.3.1) for 3D reconstruction of dendritic segments and the Spine Classifier MATLAB extension for spine subtyping (Bevan et al., 2020a; Bevan et al., 2020b). Classifications of spines were pre-determined in the software settings. Stubby spines <0.8 μm; mushroom spines >0.8 μm but <3 μm with a spine head diameter greater than neck width; thin spines >0.8 μm but <3 μm.

### 2.4 Novel object recognition

The Novel Object Recognition (NOR) tests were performed as previously described (Webberley et al., 2022; Webberley et al., 2023). The Discrimination Ratio (DR) was calculated from the time spent interacting with a novel object (NO) relative to the familiar object (FO): [DR = FO (seconds)/FO (seconds) + NO (seconds)]. A DR < 0.5 denoted little interest in the novel object (poor recognition memory) and a DR > 0.5 denoted more interest in the novel object (good recognition memory).

### 2.5 mRNA expression analysis of brain tissue

The extraction of RNA from whole brain tissue and the associated quantitative PCR (qPCR) analysis was performed as described previously (Webberley et al., 2022; Webberley et al., 2023) using intron-spanning gene-specific primers (Supplementary Table S1). mRNA expression levels were determined via the 2^−∆Ct^, where ∆Ct represents the difference between the threshold cycles (Ct) for each of the target genes and the housekeeping gene (β-actin).

### 2.6 Plasma analysis

Cytokines/chemokines [interferon-*γ* (IFN-γ), interleukin (IL)-2, IL-4, IL-5, IL-6, IL-10 and IL-1β, keratinocyte chemoattractant/growth regulated oncogene (KC/GRO) and tumour necrosis factor-*α* (TNF-α)] were measured in plasma using an MSD multiplex platform (Central Biotechnology Services, Cardiff University, United Kingdom). Plasma lipids including Total Cholesterol (TC), High-Density Lipoprotein (HDL), Low-Density Lipoprotein (LDL) and very Low-Density Lipoprotein (vLDL) were assayed using the Cholesterol assay kit and Triglycerides (TG) were assayed using the Triglyceride assay kit (Abcam, Cambridge, United Kingdom) according to manufacturer’s instructions.

### 2.7 16S gDNA preparation, sequencing and analysis

Genomic DNA extraction, library preparation, sequencing and analysis was according to (Webberley et al., 2022; Webberley et al., 2023). Briefly, gDNA was extracted using the *QIAamp® Fast DNA Stool Mini Kit* (Qiagen, Germany) according to the manufacturer’s instructions. 16S rRNA gene libraries were prepared following the *Illumina 16S Metagenomic Sequencing Library Preparation Protocol* targeting the V1–V2 regions, with the inclusion of *Bifidobacterium*-specific sequences (Moshkelgosha et al., 2021), and sequenced on the *Illumina MiSeq* platform (2 × 300 bp chemistry). Raw reads were processed with *DADA2* (Callahan et al., 2016) to obtain amplicon sequence variants (ASVs), which were taxonomically assigned against the *SILVA* database (v138.1) (Quast et al., 2012). Outputs were analysed in *phyloseq* (v1.50.0) (McMurdie and Holmes, 2013) with alpha diversity assessed using Shannon and Simpson indices, and beta diversity evaluated with Bray–Curtis dissimilarities and PERMANOVA [*Vegan adonis* (v2.7–1), 999 permutations; Oksanen et al., 2016]. Differential abundance was determined with *DESeq2* (v1.46.0) (Love et al., 2014).

### 2.8 Microbial enumeration

Faecal pellets were homogenised in Maximum Recovery Diluent (MRD, Oxoid, United Kingdom) and decimal dilution series were set up in MRD and plated on selective growth media (Oxoid, United Kingdom; Supplementary Table S2). Results are expressed as log_10_ colony forming units (CFU)/per gram wet weight.

### 2.9 Statistical analysis

All data are presented as mean ± standard deviation (SD) of the indicated number of mice. Normality was assessed by Shapiro–Wilk test and visual inspection of histograms and Q-Q plots. Where normality was not met, data were log-transformed and normality confirmed as previously described. For single comparisons, differences between groups were assessed using two-tailed unpaired Student’s *t*-tests. For multiple group comparisons, one-way ANOVA followed by Tukey’s post-hoc test or two-way ANOVA followed by Šídák’s post-hoc test was performed. Statistical analyses were conducted using GraphPad Prism (Version 10.6.1, GraphPad Software, United States). Value of *p* < 0.05 were considered statistically significant.

## 3 Results

### 3.1 Lab4 supplementation improves neuronal spine density and novel object recognition and modulates genes involved in inflammation and oxidative defense in the brain

**Figure 1:**
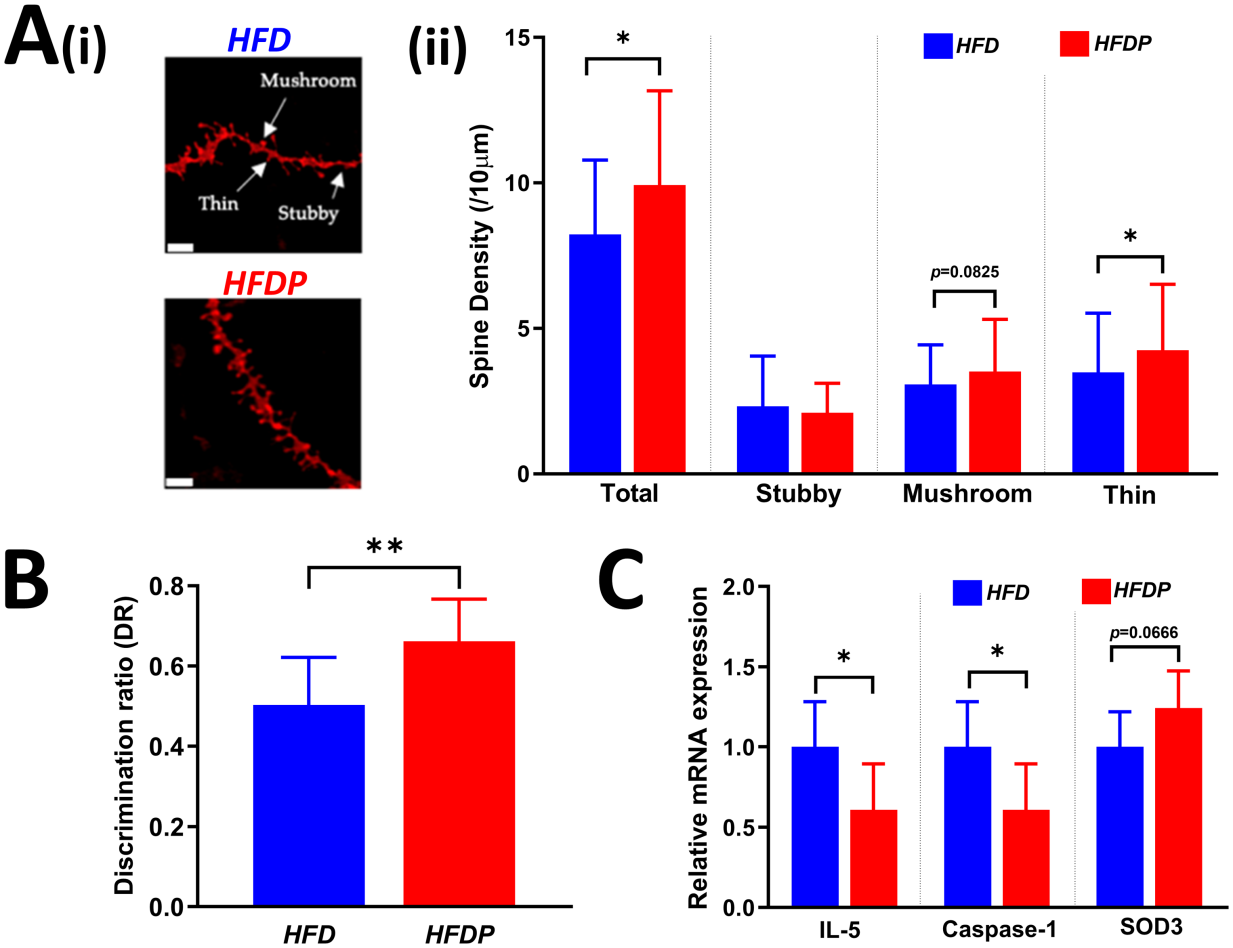
Neuronal spine density, discrimination ratio, and brain gene expression. **[A(i)]** Representative images of stubby, mushroom, and thin neuronal spines and **[A(ii)]** neuronal spine densities in the hippocampus, **(B)** discrimination ratios (DRs) as determined during NOR testing, and **(C)** whole brain mRNA expression of IL-5, Caspase-1, and SOD in the *HFD* and *HFDP* groups at the study endpoint. Data are presented as the mean ± SD of at least seven mice per group. * *p* < 0.05, ** *p* < 0.01 or as stated. HFD, high fat diet; IL-5, interleukin-5; SOD3, superoxide dismutase 3.

Between group analysis of the number of hippocampal neuronal spines indicated the number in the *HFD* (High Fat Diet) control group was significantly lower than in the *HFDP* (probiotic) group at the end of the study (8.82 ± 0.31 spines/10 μm *vs.* 9.97 ± 0.40 spines/10 μm, respectively, *p* = 0.0211, Figures 1Ai,ii). The numbers of thin spines were significantly lower in the *HFD* group (*HFD*: 3.50 spines ± 0.22/10 μm *vs. HFDP*: 4.25 ± 0.26 spines/10 μm, *p* = 0.0265, Figures 1Ai,ii) and there was a trend for lower numbers of mushroom spines in the *HFD* group (*p* = 0.0825).

Comparison of the Discrimination Ratios determined from the Novel Object Recognition (NOR) test at the end of the study indicated a significantly poorer performance for the mice in the *HFD* group (*HFD*: DR = 0.50 ± 0.04 *vs. HFDP*: DR = 0.66 ± 0.03, *p* = 0.0068, Figure 1B).

Expression levels of genes related to cognition, neurodegeneration, inflammation, apoptosis and oxidative stress were measured in whole brain extracts and revealed significantly lower levels of IL-5 and Caspase-1 and a trend toward higher expression of SOD3 in the *HFDP* group compared to the *HFD* group (Figure 1C). For IL-5: *HFD* = 1.00 ± 0.48 *vs.* 0.54 ± 0.025 in *HFDP* (*p* = 0.0445); for Caspase-1: *HFD:* 1.00 ± 0.28 *vs. HFDP*: 0.61 ± 0.29 (*p* = 0.0241) and for SOD3: *HFD:* 1.00 ± 0.22 *vs. HFDP*: 1.24 ± 0.23 (*p* = 0.0666). No other gene changes were observed (Supplementary Table S2).

### 3.2 Lab4 supplementation dampens systemic inflammation

Circulating concentrations of cytokines/chemokines after supplementation were quantified and plasma levels of *TNF*-*α* were significantly lower in the *HFDP* mice compared to the *HFD* mice (*p* = 0.0387, Table 1).

**Table 1:** Plasma cytokine/chemokine concentrations (pg/ml) at endpoint.

	*HFD* group	*HFDP* group	*p*
Tumor necrosis factor-*α*	21.66 ± 7.26	16.18 ± 3.23	0.0387
Interleukin-1β	1.59 ± 0.52	2.02 ± 1.48	0.7064
Interleukin-2	0.73 ± 0.40	0.65 ± 0.28	0.6828
Interleukin-4	0.12 ± 0.10	0.23 ± 0.29	0.5043
Interleukin-5	5.12 ± 4.61	4.62 ± 3.10	0.8752
Interleukin-6	24.18 ± 11.24	44.54 ± 49.01	0.6162
Interleukin-10	117.90 ± 71.97	77.12 ± 29.84	0.1230
Interferon-*γ*	0.20 ± 0.08	0.23 ± 0.07	0.3801
Keratinocyte chemoattractant/growth regulated oncogene	400.98 ± 277.53	341.8 ± 236.64	0.5188

Data are expressed as the mean ± SD from at least six mice per group.

### 3.3 Lab4 impacts on weight gain in 3xTg-AD mice

**Figure 2:**
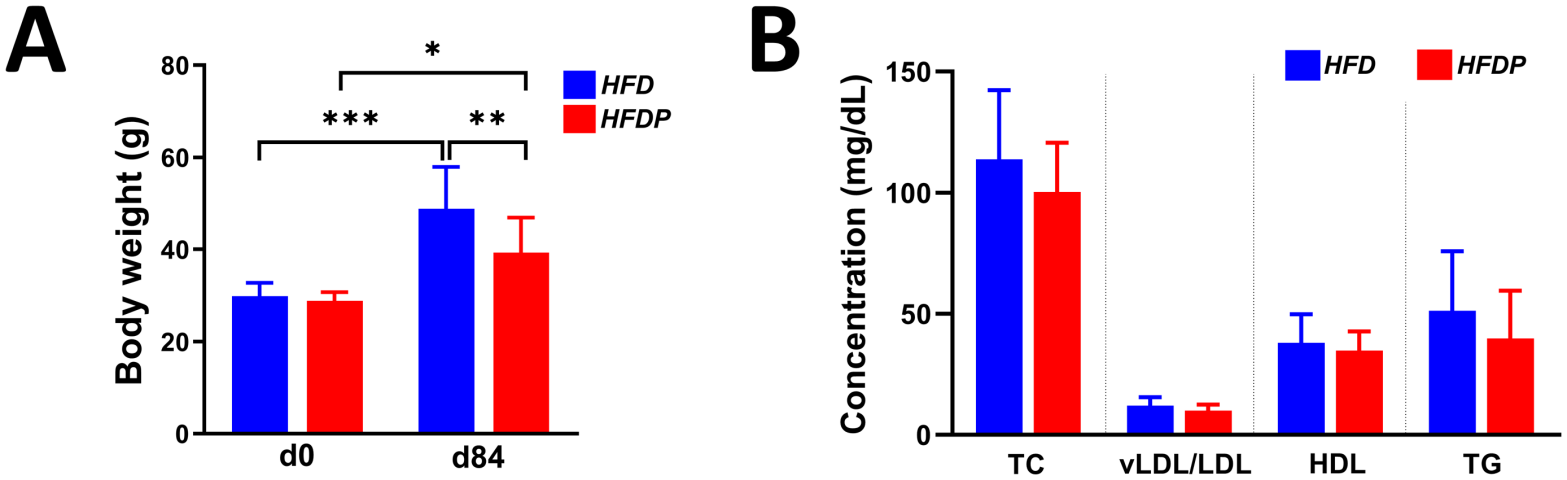
Body weight and plasma lipids. Changes in **(A)** absolute body weight over the duration of the study and **(B)** plasma lipids at endpoint in the *HFD* and *HFDP* groups. Data are expressed as mean ± SD of at least nine mice per group. * *p* < 0.05, ** *p* < 0.01 or *** *p* < 0.001. HFD, high fat diet; d, day; TC, total cholesterol, LDL/LDL, very low density lipoprotein/low density lipoprotein; HDL, high density lipoprotein; TG, triglycerides.

The group average body weights for the mice were comparable at baseline (28.99 ± 0.90 g in *HFD vs*. 28.83 ± 0.63 g in *HFDP*) but at day 84 the *HFDP* mice were significantly lighter than the *HFD* mice (39.27 ± 2.54 g *vs.* 48.81 ± 2.88 g, respectively, *p* = 0.0121, Figure 2A). By day 84, body weights in both groups had significantly increased from baseline, by 63.4% (*p* < 0.0001) for *HFD* and by 36.2% (*p* = 0.0067) for *HFDP*.

Analysis of plasma lipids showed that concentrations of total cholesterol, vLDL/LDL, HDL and triglycerides were similar between groups at endpoint (Figure 2B).

### 3.4 Lab4 supplementation modulated the composition of the faecal microbiota

**Figure 3:**
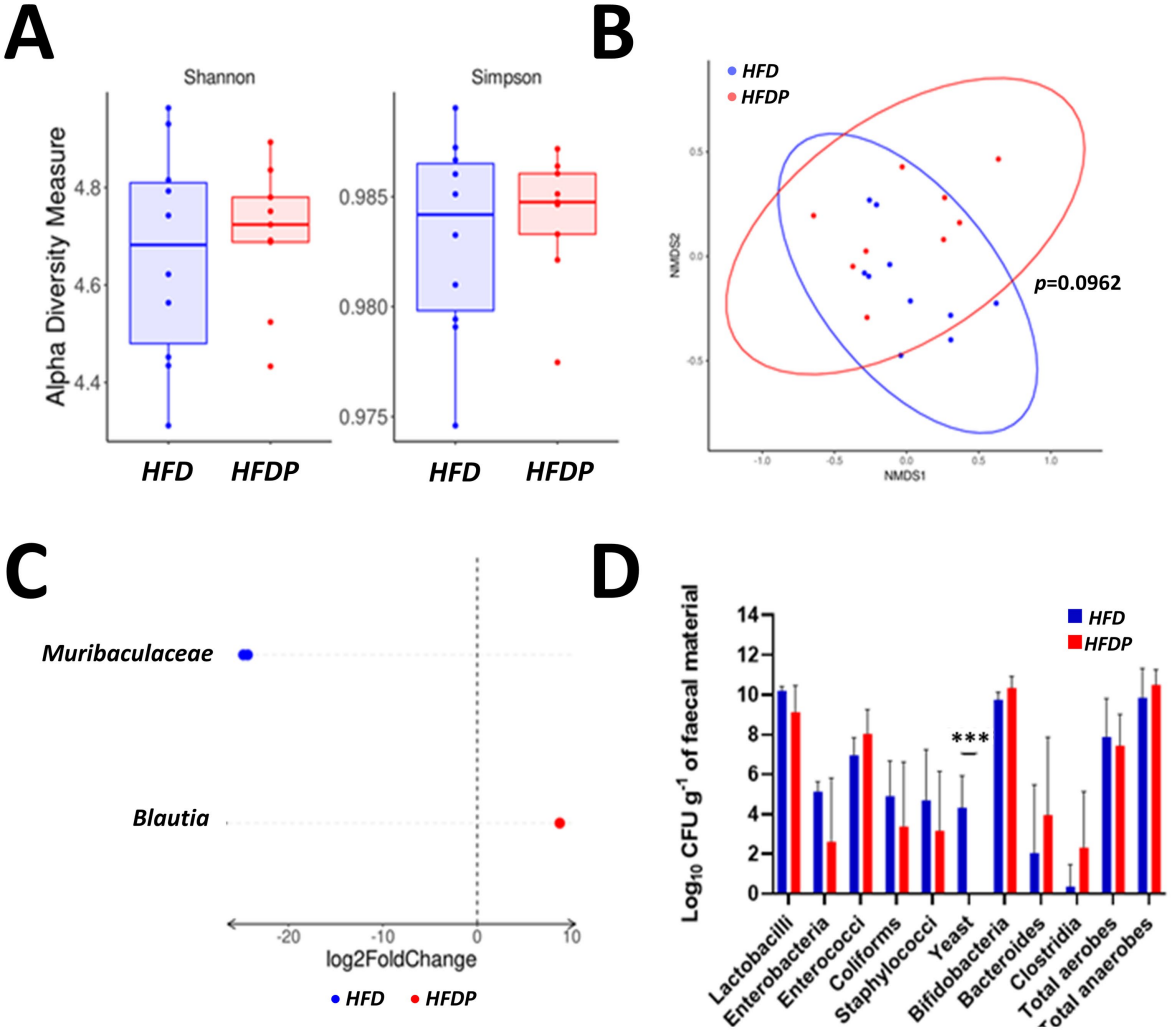
The faecal microbiota. **(A)** Alpha diversity indices, **(B)** beta-diversity non-metric multidimensional scaling (NMDS), **(C)** differentially abundant bacterial species, and **(D)** viable numbers of common gut bacteria in the *HFD* and *HFDP* groups at the study endpoint. Data are presented for at least nine mice per group. ***p < 0.001 or as stated.

At the end of the study the faecal microbiota alpha diversity (Shannon and Simpson indices; Figure 3A) was similar between the groups, but with a trend towards different microbiota compositions (*p* = 0.0962, Figure 3B). Differential abundance analysis revealed a lower abundance of the *Muribaculaceae* family (*p* < 0.0001, Figure 3C) and a higher abundance of the *Blautia* genus (*p* = 0.0042) in the *HFDP* group relative to the *HFD* group. When cultured on selective growth media the faecal samples of the *HFDP* group contained less viable yeast than the *HFD* group (*p* < 0.0001, Figure 3D), but no other significant differences were detected.

## 4 Discussion

In this study exploring the impact of the Lab4 probiotic on neurodegeneration in a challenged (high fat diet fed) 3xTg AD mouse model, the probiotic fed mice had higher densities of neuronal spines in the hippocampus, a better level of cognition, differences in the inflammatory status in the brain and circulation, less diet induced weight gain and evidence of differences in the composition of the faecal microbiota compared with the mice fed high fat diet alone.

Feeding a high fat diet has been used as a means of accelerating neurodegeneration in mice (Webberley et al., 2023) and is associated with the loss of the neuronal spines that are responsible for the formation and maintenance of the integrity of the synaptic connections that underlie all cognitive functions (Gadhave et al., 2024). We found higher densities of the hippocampal thin neuronal spines in the mice receiving the Lab4 probiotic and these are considered important for learning and new memory formation (Tackenberg et al., 2009). We have seen similar results with similar probiotic formulations in high fat diet challenged 3xTg-AD mice (Webberley et al., 2022; Webberley et al., 2023) and shown that Lab4 can impart neuroprotective effects on human SHSY5Y neurones *in vitro* (Michael et al., 2019).

The mice in the *HFDP* group performed significantly better during the novel object recognition test compared to the *HFD* group, supporting the presence of the higher numbers of the “learning and memory” thin dendrites in the hippocampus (Burdakov and Peleg-Raibstein, 2020). Memory preservation associated with the Lab4 probiotic has also been seen in aged Wister rats (O'Hagan et al., 2017) and these findings add to the growing pool of evidence supporting the ability of probiotic bacteria to improve novel object recognition in 3xTg-AD mice (Bonfili et al., 2017; Webberley et al., 2022; Webberley et al., 2023; Bonfili et al., 2025).

There was less gene expression of the pro-inflammatory mediators IL-5 and Caspase-1 that are linked with neurodegeneration and synaptic dysfunction (Tennakoon et al., 2022; Wang et al., 2024), suggesting an anti-inflammatory activity for the probiotic and this is further supported by the significantly lower levels of TNF-*α* which is a potent pro-inflammatory cytokine strongly associated with neurodegeneration and cognitive decline (Lo and Zeng, 2025). The anti-inflammatory capabilities of Lab4 have been observed previously, including the reduction of circulating TNF-α in Lab4 fed Wistar rats (Webberley et al., 2021) and the reduction of circulating pro-inflammatory IL-6 in human subjects (Mullish et al., 2023), most likely linked to its ability to increase circulating levels of anti-inflammatory short chain fatty acids (SCFAs) such as butyrate (Michael et al., 2025).

Oxidative stress and the accumulation of amyloid (Aβ)/tau deposits are also recognised drivers of neurodegeneration (Karapetyan et al., 2022; Houldsworth, 2024). In the brains of the *HFDP* mice there was indication of increased expression of SOD3, an enzyme involved in the conversion of toxic superoxide radicals into hydrogen peroxide and oxygen (Chidambaram et al., 2024) and similar results have been seen in human SHSY5Y neurones where Lab4 enhanced the expression of key genes involved in anti-oxidant defense (Michael et al., 2019). Amyloid/tau deposits can hinder synaptic communication and trigger inflammation and neuronal death (Hampel et al., 2021) but there was an absence of amyloid/tau pathology in the 3xTg mice. In previous studies, 3xTg mice from the same community fed high fat diet failed to show any evidence of amyloid/tau pathology (Webberley et al., 2022; Webberley et al., 2023). It has been recognised that phenotypic changes can occur in sublines of 3xTg mice (Belfiore et al., 2019).

Obesity increases the risk of development of neurodegenerative diseases such as AD and Parkinson’s disease (Kueck et al., 2023). The high fat diet feeding was primarily employed to accelerate neurodegeneration in the 3xTg mice but it has also provided an opportunity to compare the potential for any metabolic impact of Lab4. We observed less weight gain in the *HFDP* group adding to the pool of evidence supporting the beneficial metabolic effects of probiotic bacteria (Guo et al., 2025) but the lowered weight gain did not occur alongside changes in plasma lipid profile; the Lab4 probiotic has been shown to impair cholesterol transport *in vitro* (Michael et al., 2017) and reduce circulating levels of low-density lipoprotein (LDL) in healthy Wistar rats (Webberley et al., 2021).

Obesity is linked with a dysbiotic composition of the gut microbiota (Van Hul and Cani, 2023) and the faecal microbiota was assessed by both 16s sequencing and traditional plate culture techniques (‘culturomics’). The probiotic did not impact the alpha-diversity of the groups but there was a trend towards a significant shift in composition (beta-diversity). Compared to the *HFD* group, the *HFDP* group had a higher prevalence of *Blautia* and a lower prevalence of *Muribaculaceae*. Low abundance of *Blautia* species, important SCFA producers, has been observed during neurodegeneration (Liu et al., 2024) and high prevalence of *Muribaculaceae* has been linked with disease progression in AD mice (Kundu et al., 2021). Traditional culture of the mouse faeces indicated significantly less viable yeast in the *HFDP* group compared to the *HFD* control, consistent with previous findings in high fat diet fed 3xTg mice (Webberley et al., 2022) and a high abundance of commensal yeast in the gut has been linked to the progression of both AD (Alonso et al., 2014; Pisa et al., 2015a; Pisa et al., 2015b; Wu et al., 2019) and obesity (García-Gamboa et al., 2021). Future work will include the collection of baseline faecal samples in order to assess the longitudinal impact of the probiotic upon the composition of the microbiota during AD development.

In summary, this study has demonstrated differences in cognition, neurodegeneration and the composition of the gut microbiota in 3xTg-AD mice and 3xTg-AD mice receiving the Lab4 probiotic whilst metabolically challenged by means of a high fat diet. The mice receiving the probiotic had lower abundance of the AD associated *Muribaculaceae* compared to the controls. Further work is needed to assess changes from baseline and to gain an understanding of the mechanisms of action, particularly in relation to amyloid/tau pathology.

## Data Availability

The datasets presented in this study can be found in online repositories. The names of the repository/repositories and accession number(s) can be found at: https://www.ebi.ac.uk/ena, PRJEB51991.
