# Supplementary material for: The impact of the Lab4 probiotic on neurodegenerative processes in a murine Alzheimer’s disease model
Source: Front Neurosci. 2026 Mar 31;20:1791299. doi: 10.3389/fnins.2026.1791299 (PMC13076349; doi:10.3389/fnins.2026.1791299)
Supplement: Supplementary file 1 [file Table_1.DOCX]

**Supplementary Material: The impact of the Lab4 probiotic on neurodegenerative processes in a murine Alzheimer’s Disease model**

**Supplementary Table S1.** Oligonucleotide sequences for primers used in RT-qPCR of whole brain tissue and mRNA expression levels of genes related to neurodegeneration, cognition, inflammation, apoptosis and oxidative defence.

| **Gene function** | **Gene name** | **Abbreviation** | **Forward primer**  **(5'-3')** | **Reverse primer**  **(5'-3')** | **Fold change in *HFD-Lab4* group in relation to *HFD* group:** | | ***p* value** |
| --- | --- | --- | --- | --- | --- | --- | --- |
|  |  |  |  |  | **Mean** | **SD** |  |
| Neurodegeneration-related | Amyloid beta precursor protein | APP | GACCACTCGACCAGGTTCTG | ACACCAGTTTTTGATGGCGG | 1.07 | 0.16 | 0.4977 |
|  | Presenilin 1 | PSEN-1 | TATACCCGGAAGGACGGTCA | CAGGCGTGGATGACCTTGTA | 1.13 | 0.17 | 0.2624 |
| Cognition/synaptic function | Tryptophan hydroxylase 2 | TPH2 | GGACGGAGGAAGAAGATCTCG | TAAATCCTGGATGGTCGGCA | 1.82 | 1.43 | 0.1645 |
|  | Amyloid beta precursor protein | APP | GACCACTCGACCAGGTTCTG | ACACCAGTTTTTGATGGCGG | 1.07 | 0.16 | 0.4977 |
|  | Brain-derived neurotrophic factor | BDNF | GGGTCACAGCGGCAGATAAA | GCCTTTGGATACCGGGACTT | 1.07 | 0.29 | 0.5623 |
|  | Complexin 1 | CPLX1 | GACCCCGATGCTGCTAAGAA | TGCCATACTTGTCTCTTATACCCT | 1.00 | 0.36 | 0.9861 |
|  | Complexin 2 | CPLX2 | AGTGGCTTAGACGGTTGCTG | TGGTGGCCCCTCCGA | 1.26 | 0.44 | 0.2043 |
|  | Glutamate ionotropic receptor NMDA type subunit 1 | GRIN1 | GTCCAGCGTCTGGTTTGAGA | TTCTCTGCCTTGGACTCACG | 1.12 | 0.23 | 0.6965 |
|  | Glutamate ionotropic receptor NMDA type subunit 2B | GRIN2B | GAACCTCCTGTGTGAGAGGAAAT | CTGGATGCCGGGGATAGAAAG | 1.12 | 0.65 | 0.6639 |
|  | Glutamate ionotropic receptor AMPA type subunit 1 | GRIA1 | CCCTGAGAGGTCCCGTAAAC | GCTCAGAGCACTGGTCTTGT | 1.03 | 0.47 | 0.9016 |
|  | NMDA receptor synaptonuclear signalling and neuronal migration factor | NSMF | TGATGTCCCCATCCGTACCT | CCCCTGAATACCGAGATGGC | 1.02 | 0.35 | 0.8983 |
| Inflammation | Interleukin 1 beta | IL-1β | TGCCACCTTTTGACAGTGATG | TGATGTGCTGCTGCGAGATT | 0.92 | 0.40 | 0.6968 |
|  | Tumour necrosis factor alpha | TNF-α | GTCCCCAAAGGGATGAGAAGT | TGGTTTGCTACGACGTGGG | 0.84 | 0.33 | 0.4523 |
|  | Interleukin 4 | IL-4 | ACAGGAGAAGGGACGCCAT | CTGTGGTGTTCTTCGTTGCTG | 0.75 | 0.31 | 0.3864 |
|  | Interleukin 5 | IL-5 | AGCAATGAGACGATGAGGCTT | TCCTCGCCACACTTCTCTTTT | 0.54 | 0.25 | 0.0445 |
|  | Interleukin 6 | IL-6 | GAGGATACCACTCCCAACAGACC | AAGTGCATCATCGTTGTTCATACA | 1.12 | 0.21 | 0.5116 |
|  | Interleukin 18 | IL-18 | CAAGTTTACAAGCATCCAGGCA | CATTGTTCCTGGGCCAAGAGG | 0.88 | 0.16 | 0.2311 |
|  | Interleukin 10 | IL-10 | TAAGGGTTACTTGGGTTGCCA | GAGAAATCGATGACAGCGCC | 0.54 | 0.29 | 0.2633 |
|  | Keratinocyte-derived chemokine / Growth-regulated oncogene | KC-GRO | ACCGAAGTCATAGCCACACTC | ACTTGGGGACACCTTTTAGCA | 1.48 | 1.46 | 0.4266 |
| Inflammasome/pryroptosis | Caspase 1 | Caspase-1 | CTATGGACAAGGCACGGGAC | TCAGCTGATGGAGCTGATTGA | 0.61 | 0.29 | 0.0241 |
|  | NLR family pyrin domain containing 3 | NLRP3 | GACACGAGTCCTGGTGACTT | GGCTTAGGTCCACACAGAAAG | 1.04 | 0.47 | 0.8542 |
|  | NLR family pyrin domain containing 1a | NLRP1a | CATAGAGGAGCAGGCAGGTCTC | TGCTTCAAGACGCTCTTCTGT | 1.07 | 0.35 | 0.6802 |
|  | NLR family pyrin domain containing 1b | NLRP1b | TCCAGTGGAAGAGTGGGGTCT | GGAGCTCTCGGATACTGCTTC | 1.01 | 0.25 | 0.9618 |
| Apoptosis | BCL2 associated X protein | BAX | TGCAGAGGATGATTGCTGAC | GATCAGCTCGGGCACTTTAG | 1.01 | 0.10 | 0.9496 |
|  | B-cell lymphoma 2 | BCL-2 | TGAGTACCTGAACCGGCATCT | GCATCCCAGCCTCCGTTAT | 1.18 | 0.21 | 0.1553 |
|  | BAX/Bcl-2 ratio | BAX/BCL-2 | NA | NA | 0.88 | 0.19 | 0.2804 |
| Oxidative defence | Superoxide dismutase 1 | SOD1 | CCAGTGCAGGACCTCATTTT | CACCTTTGCCCAAGTCATCT | 1.14 | 0.20 | 0.3619 |
|  | Superoxide dismutase 2 | SOD2 | GGCCAAGGGAGATGTTACAAC | GCAACTCTCCTTTGGGTTCTC | 1.12 | 0.16 | 0.2775 |
|  | Superoxide dismutase 3 | SOD3 | TTCTTGTTCTACGGCTTGCTAC | CTCCATCCAGATCTCCAGCACT | 1.24 | 0.23 | 0.0666 |
|  | Glutathione peroxidase 1 | GPx1 | CAGGAGAATGGCAAGAATGAAGAG | GGCATTCCGCAGGAAGGTAAAGAGCGG | 1.09 | 0.09 | 0.3682 |
|  | Catalase | CAT | GCGGATTCCTGAGAGAGTGGTAC | GCCTGACTCTCCAGCGACTGTGGAG | 1.03 | 0.30 | 0.8310 |
|  | Glutathione-disulphide reductase | GSR | CCAATGTCAAAGGCGTCTATG | AGACCACAGTAGGGATGTTGTCA | 1.14 | 0.27 | 0.3434 |

Housekeeping gene: β-actin, Forward; 5’-ACACCCGCCACCAGTTCGCCAT-3’, Reverse; 5’-CACACCCTGGTGCCTAGGGCGGCCCACGATG-3’

# Supplementary Table S2: Selective microbial culture conditions

| **Bacteria** | **Selective Culture Medium** | **Growth Conditions** |
| --- | --- | --- |
| Lactobacilli | De Man, Rogosa and Sharpe (MRS) agar & Polymixin B (CM0361, SR0099E) | Aerobic, 37°C for 48 h |
| Enterobacteria | MacConkey No. 3 agar (CM0115B) | Aerobic, 37°C for 48 h |
| Enterococci | Kanamycin Aesculin Azide agar & Kanamycin supplement (CM0591, SR0092E) | Aerobic, 37°C for 48 h |
| Coliforms | Tergitol-7 agar (T7) & T.T.C (0.125%) supplement (CM0793B, SR0148A) | Aerobic, 37°C for 48 h |
| Staphylococci | Baird Parker agar + egg yolk tellurite emulsion supplement (CM0275B, SR0054) | Aerobic, 37°C for 48 h |
| Yeasts | Dichloran Rose Bengal Chloramphenicol (DRBC) agar & chloramphenicol supplement (CM0727, SR0078E) | Aerobic, 37°C for 48 h |
| Bifidobacteria | MRS-X agar^1^ & defibrinated sheep blood (SR0051C) | Anaerobic^2^, 37°C for 48 h |
| Bacteroides | Wilkins-Charun-in lgren Anaerobe agar & G-N Anaerobe Selective supplement (CM0619, SR0108) | Anaerobic^2^, 37°C for 48 h |
| *Clostridia* | *Clostridium difficile* agar & Clostridium difficile supplement (CM0601B, SR0096) | Anaerobic^2^, 37°C for 48 h |
| Total aerobes | Colombia & 5% horse blood agar (HBA) (Biomerieux, 43050) | Aerobic, 37°C for 48 h |
| Total anaerobes | HBA | Anaerobic^2^, 37°C for 48 h |

^1^MRS agar supplemented with 0.25 g/l L-cysteine hydrochloride monohydrate, 1 g/l Lithium chloride and 1.5 g/l Sodium propionate.

^2^Anaerobic conditions: 10% carbon dioxide, 10% hydrogen, 80% nitrogen.
